# The impact of right ventricular injury on the mortality in patients with acute respiratory distress syndrome: a systematic review and meta-analysis

**DOI:** 10.1186/s13054-021-03591-9

**Published:** 2021-05-21

**Authors:** Ryota Sato, Siddharth Dugar, Wisit Cheungpasitporn, Mary Schleicher, Patrick Collier, Saraschandra Vallabhajosyula, Abhijit Duggal

**Affiliations:** 1 Department of Critical Care Medicine, Respiratory Institute, Cleveland Clinic, 9500 Euclid Avenue, Cleveland, OH USA; 2 Cleveland Clinic Lerner College of Medicine, Case Western University Reserve University, Cleveland, OH USA; 3 Division of Nephrology and Hypertension, Department of Medicine, Mayo Clinic, Rochester, MN USA; 4 The Cleveland Clinic Floyd D. Loop Alumni Library, Cleveland Clinic, Cleveland, OH USA; 5 Department of Cardiovascular Medicine, Heart, Vascular, and Thoracic Institute, Cleveland Clinic, Cleveland, OH USA; 6 Department of Cardiovascular Medicine, Mayo Clinic, Rochester, MN USA; 7 Division of Pulmonary and Critical Care Medicine, Department of Medicine, Mayo Clinic, Rochester, MN USA; 8 Center for Clinical and Translational Science, Mayo Clinic Graduate School of Biomedical Sciences, Rochester, MN USA; 9 Section of Interventional Cardiology, Division of Cardiovascular Medicine, Department of Medicine, Emory University of School of Medicine, Atlanta, GA USA

**Keywords:** Right ventricular dysfunction, Acute cor pulmonale, Acute respiratory distress syndrome, Acute lung injury

## Abstract

**Background:**

Previous studies have found various incidences of right ventricular (RV) injury and its association with clinical outcome in patients with acute respiratory distress syndrome (ARDS). In this systematic review and meta-analysis, we aimed to investigate the impact of the presence of RV injury on mortality in patients with ARDS.

**Method:**

We searched Medline, Embase, and the Cochrane Central Register of Controlled Trials for studies investigating the association between RV injury and mortality. Two authors independently evaluated whether studies meet eligibility criteria and extracted the selected patients and studies characteristics and outcomes. RV injury was diagnosed by trans-thoracic echocardiogram (TTE), trans-esophageal echocardiogram (TEE) and PAC (pulmonary artery catheter) in the included studies. The primary outcome was the association between mortality and the presence of RV injury in patients with ARDS. The overall reported mortality was defined as either the intensive care unit (ICU) mortality, in-hospital mortality, or mortality within 90days, and short-term mortality was defined as ICU-mortality, in-hospital mortality, or mortality within 30days.

**Results:**

We included 9 studies (*N*=1861 patients) in this meta-analysis. RV injury that included RV dysfunction, RV dysfunction with hemodynamic compromise, RV failure, or acute cor-pulmonale was present in 21.0% (391/1,861). In the pooled meta-analysis, the presence of RV injury in patients with ARDS was associated with significantly higher overall mortality (OR 1.45, 95% CI 1.131.86, *p*-value=0.003, *I*^2^=0%), as well as short-term mortality (OR 1.48, 95% CI 1.141.93, *p*-value=0.003, *I*^2^=0%).

**Conclusion:**

In this systematic review and meta-analysis including 1861 patients with ARDS, the presence of RV injury was significantly associated with increased overall and short-term mortality.

*Trial registration*: The protocol was registered at PROSPERO (CRD42020206521).

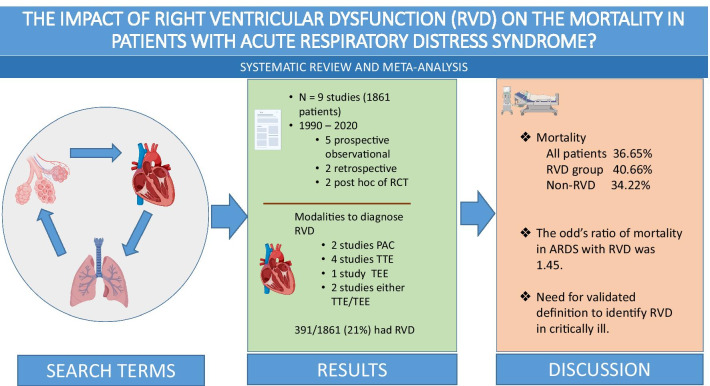

**Supplementary Information:**

The online version contains supplementary material available at 10.1186/s13054-021-03591-9.

## Background

Despite advances in the management of acute respiratory distress syndrome (ARDS) including lung-protective ventilation, prone positioning, and neuromuscular blockade, the mortality still remains alarmingly high, with a recent meta-analysis reporting a mortality of 3040% [1]. There is evolving evidence that right ventricular (RV) injury with associated hemodynamic compromise might be a significant factor associated with higher mortality in ARDS [2].

The etiology of RV injury in patients with ARDS is complex and is driven primarily by an increase in pulmonary vascular resistance due to ongoing inflammation, hypoxemia-driven vasoconstriction, micro-thrombi formation, and vascular remodeling [3]. The thin-walled right ventricle with a low contractile reserve is ill-adapted for an abrupt increase in afterload, and this leads to acute cor-pulmonale in these patients. RV injury is further exacerbated with the use of positive pressure ventilation in patients with ARDS due to increased RV afterload from increased intrathoracic pressure [4].

Historically, pulmonary artery catheters (PAC) were used to evaluate right heart function in ARDS patients, however, contemporary intensive care units (ICU) rarely use the PAC in routine practice [5]. The widespread usage of critical care echocardiography in recent times has renewed interest in better understanding not only the prognostic role of RV injury in mortality associated with ARDS but also the factors associated with RV injury [2]. Previous studies have reported a wide range of the prevalence of RV injury in ARDS. Also, most of these studies had small sample sizes and varying methodologies which led to discordant results. In this systematic review and meta-analysis, we aim to pool these studies to better understand the prevalence of RV injury and to report on the mortality in patients with ARDS who develop RV injury.

## Methods

### Protocol

This study complied with the Preferred Reporting Items for Systematic Reviews and Meta-Analysis (PRISMA) statement [6, 7], and the Meta-Analyses of Observational Studies in Epidemiology proposals [8]. Our protocol was registered at PROSPERO (CRD42020206521).

### Search strategy

A comprehensive search of Medline, Embase, and Cochrane Central Register of Controlled Trials was conducted with the search strategy detailed in Additional file 1. The search period was limited from 1990 to 2020. Our search was updated on August 28, 2020.The characteristics of each studyis described in Table 1.

**Table 1:** Characteristics of each study

Authors	Country	Sample size	Setting	Study period	Definition of ARDS	Definition of RV injury	Mortality
Osman /2009	France	145	Multi-center, post-hoc analysis of RCT	January 1999June 2001	American-European consensus conference	(1) MPAP>25mmHg, (2) CVP>PAOP, and (3) SVI<30mL/m^2^, based on PAC	28-day
Bull/2010	United States	367	Post-hoc analysis of multicenter randomized controlled trial	June 2000Oct 2005	American-European consensus conference	CVP>PAOP	60-day
Fichet/2012	France	50	Single-center, prospective	Not reported	American-European consensus conference	TAPSE<12mm or St<11.5cm/sec	ICU
Legras/2015	France	166	Multi-center, prospective	November 2009-June 2012	American-European consensus conference	RVEDA/LVEDA ratio>0.6 associated with systolic paradoxical ventricular septal motion by TTE or TEE	28-day
Lazzeri/2016	Italy	74	Single-center, retrospective	October 2009December 2013	Berlin definition. All included patients underwent VV-ECMO	RVEDA/LVEDA ratio>0.6 by TTE or TEE	ICU
Mekonstso Dessap/2016	France	752	Multi-center, prospective	19942012	Berlin definition (Although the study was initiated begore 2011, all met the Berlin definition.)	RVEDA/LVEDA ratio>0.6 associated with septal dyskinesia by TEE	In-hospital
See/2017	Singapore	234	Single-center, prospective	September 2012May 2014	Berlin definition	RVEDA/LVEDA ratio1 by TTE	In-hospital
Bonizzoli/2018	Italy	28	Single-center, retrospective	January 2016June 2017	Berlin definition	RV free wall strain<20%	ICU
Zeiton/2018	Egypt	45	Single-center, prospective	June 2016December 2016	Berlin definition	RVEDA/LVEDA ratio>0.6 associated with septal dyskinesia by TTE	28-day

RV, right ventricle/right ventricular; ARDS, acute respiratory distress syndrome; TAPSE, tricuspid annular plane systolic excursion; St, peak systolic velocity at the tricuspid valve; ACP, acute cor pulmonale; VV-ECMO, veno-venous extracorporeal membrane oxygenation; MPAP, mean pulmonary artery pressure; TTE, transthoracic echocardiography; TEE, transesophageal echocardiography; RCT, randomized controlled trial; PAC, pulmonary artery catheter; MPAP, mean pulmonary artery occlusion pressure; CVP, central venous pressure; PAOP, pulmonary artery occlusion pressure; SVI, stroke volume index

### Study selection

We stored citations and removed duplicates using EndNote (Thomson Reuters, Toronto, Ontario, Canada). Two reviewers (R.S. and S.D.) independently reviewed the titles and abstracts obtained by the search and selected those that fit the inclusion criteria. We then retrieved these articles, independently read the full-text, and evaluated whether the articles fit our inclusion criteria on Covidence (https://www.covidence.org). When there were disagreements between the two reviewers, it was discussed with the third reviewer (S.V.) in detail to reach a consensus.

### Inclusion and exclusion criteria

Inclusion criteria were as follows: (1) Study design: interventional and observational studies; (2) Patient population: patients (18years old) with ARDS who underwent RV assessment with either transthoracic or transesophageal echocardiography (TTE or TEE), or the PAC. (ARDS was diagnosed based on either the American European consensus conference [9], or the Berlin definition [10].)

We excluded studies where a 22 table between RV function and mortality could not be constructed, conference proceedings (due to high risk of bias), and articles not written in English. If studies had duplication of data, and the same data was published at different time points, we chose the most relevant study as the representative sample for this meta-analysis.

### Data extraction

Two authors (R.S. and S.D.) independently extracted the following data from the eligible studies: year of publication, country, number of participants, mean/median age, sex, the definition of RV injury, cause of ARDS, the mortality, and inclusion and exclusion criteria.

### Outcomes

The primary outcome for this study was the overall reported mortality defined as either the intensive care unit (ICU) mortality, in-hospital mortality, or mortality within 90days. We also performed the pooled analysis for short-term mortality (ICU-mortality, in-hospital mortality, or mortality30days) and long-term mortality (>30days), as well as the pooled analysis for adjusted odds ratio for the mortality.

### Statistical analysis

The pooled odds ratios (ORs) and 95% confidence intervals (CI) were calculated using the random effect (DerSimonian- Laird) method [11]. Q statistic test, as well as *I*^*2*^ statistic with 95% CI, were used to assess heterogeneity. For Q statistic, substantial heterogeneity was defined as *p*<0.05. The *I*^2^ statistic ranges from 0 to 100% (*I*^2^<25%: low heterogeneity, *I*^2^=2550%: moderate heterogeneity, and *I*^2^>50%: substantial heterogeneity) [12].

To assess publication bias, we created the funnel plots and tested the symmetry of the funnel plots using Eggers regression test(Additional file 2) [13].

Statistical analysis was performed using Comprehensive Meta-analysis version 3 software (Biostat Inc, Eaglewood, MJ, USA) and Review Manager (RevMan) 5.4.1 software (Cochrane Information Management System).

### Assessment of the risk of bias

The risks of bias were independently evaluated by two authors (R.S. and S.D.) and verified by another author (S.V.). If there were disagreements, a discussion with the research team was held to reach a consensus. We assessed the study quality of each article using the quality of the study using a modified version of the NewcastleOttawa quality assessment scale [14].

## Results

### Search results

**Fig. 1:**
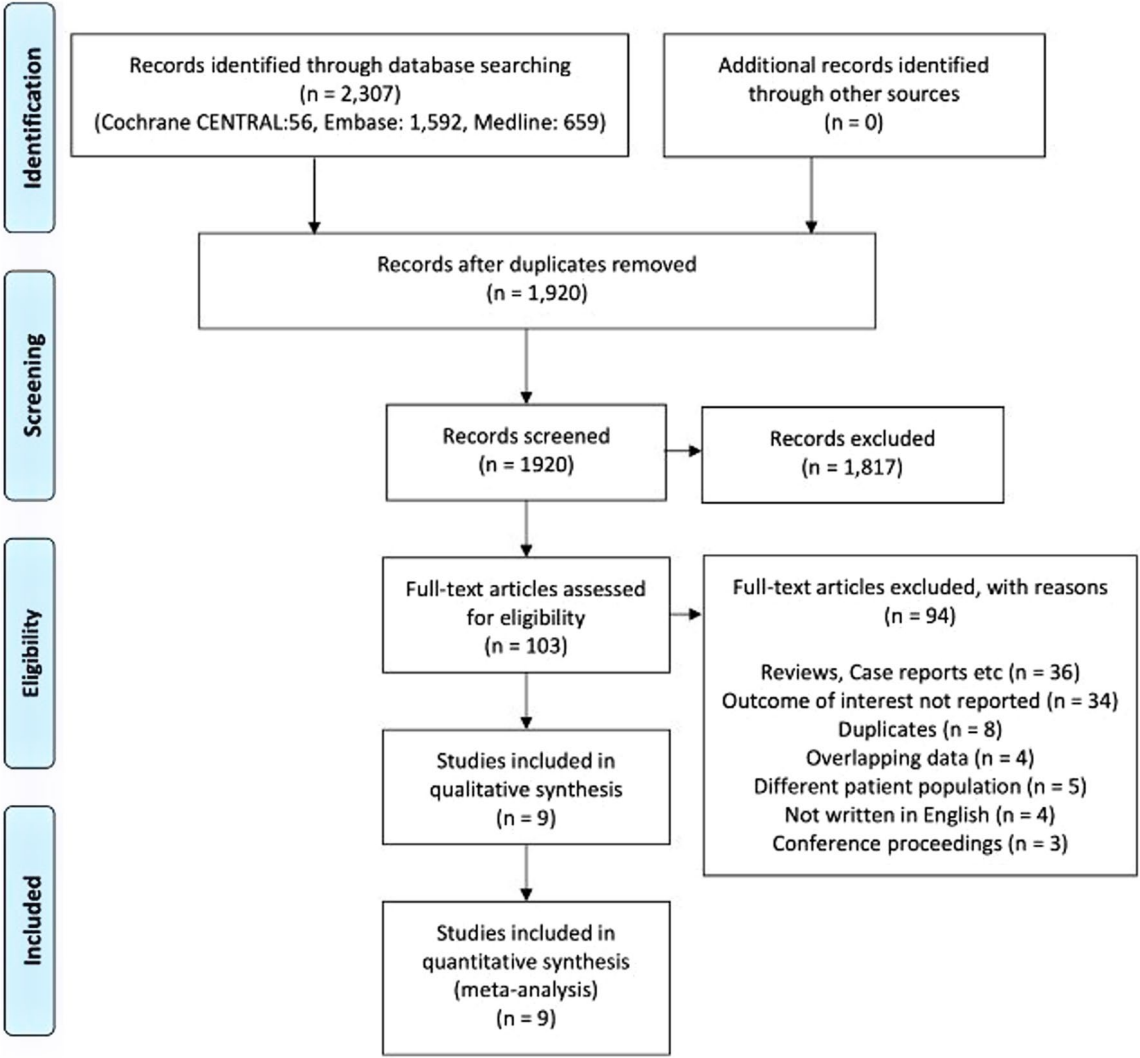
Preferred reporting items for systematic reviews and meta-analyses (PRISMA) chart. Identification and selection of studies for inclusion

Our search strategy identified 2,307 articles. After removing the duplicates and clearly irrelevant studies, full texts of 103 studies were assessed for eligibility. Fourteen studies reported the outcomes of interest for RV injury in patients with ARDS [15,28]. Nine studies with a total of 1,861 patients were included for the final analysis as shown in Fig.1 [16, 19,21, 23, 24, 26,28].

### Baseline characteristics

All articles were published between 2009 and 2018. Six studies were conducted in Europe [16, 19,21, 23, 28], one in the United States [27], and two in Asia [24, 26]. Five were prospective observational studies [20, 21, 24, 26, 28], two were retrospective studies [16, 19], and two were the post-hoc analysis of a previously conducted randomized controlled trial [23, 27] (Table 1). Inclusion and exclusion criteria for the studies are shown in Additional file 3. The risk of bias for the included studies was evaluated using a modified version of the NewcastleOttawa Scale, as shown in Table 2.

**Table 2:** NewcastleOttawa Scale assessment of pooled studies

Study	Selection				Comparability	Outcomes			Total
	Representativeness of exposed cohort	Selection of nonexposed cohort	Ascertainment of exposure	Outcome not present at the start of the study		Assessment of outcomes	Length of follow-up	Adequacy of follow-up	
Osman/2009	*	*	*	*		*	*	*	7
Bull/2010	*	*	*	*		*	*	*	7
Fichet/2012	*	*	*	*		*	*	*	7
Legras/2015	*	*	*	*		*	*	*	7
Lazzeri/2016	*	*	*	*		*	*	*	7
Mekontso Dessap/2016	*	*	*	*	**	*	*	*	9
See/2017	*	*	*	*	**	*	*	*	9
Bonizzoli/2018	*	*	*	*		*	*	*	7
Zeiton/2018	*	*	*	*		*	*	*	7

Although multivariate analysis was performed, it was not for 28-day mortality (it was for 90-day mortality)

The mean/median age of included patients ranged from 41 to 62, and 44.473% were males. Mean/median Simplified Acute Physiology Score II score ranged from 43 to 50 (patients with RV injury: 4755, patients without RV injury: 4354). In included population, 98.5% (1,834/1,861), and 55.3% (1,030/1,861) received mechanical ventilation, and vasopressors, respectively. Eight of nine studies reported mean/median positive end-expiratory pressure (PEEP) level (range: 7 to 12.7 cmH_2_O) and P/F ratio (range: 99171) when patients were evaluated for RV injury. Plateau pressure was reported in 6 studies and it ranged from 21 to 33.6 cmH_2_O (Table 3). The definition of RV injury used in each study is reported in Table 1.

**Table 3:** The characteristics of included patients

Authors	Age	Male (%)	Fluid in 24h (ml)	Vasopressors	Mechanical ventilation	P/F ratio	PEEP (cmH_2_O)	Plateau pressure (cmH_2_O)	Compliance (mL/cmH_2_O)	LV function	SAPS II	Causes of ARDS
*Osman/2009*												
RV INJURY (+)	64 (13)	35.7% (5/14)		78.6% (11/14)	100% (14/14)	115 (26)	6 (4)	28 (6)	23 (5)	SVI: 23 (3) (mL/m^2^)	55 (25)	Extra-pulmonary ARDS (1/14) Other etiologies were not reported
RV INJURY ()	60 (16)	70.2% (92/131)		76.3% (100/131)	100% (131/131)	98 (35)	7 (4)	25 (6)	32 (10)	SVI: 36 (12) (mL/m^2^)	50 (19)	Extra-pulmonary ARDS (29/131) Other etiologies were not reported
*Bull/2010*												
RV INJURY (+)	50	55.2% (262/475)		36.4% (173/475)	100% (44/44)	160	9.3	26.2		Survived: CI: 4.5 (1.4) Died: CI; 4.4 (1.6)	APACHE III: 94.3	Not reported
RV INJURY ()					100% (323/323)							
*Fichet/2012*												
RV INJURY (+)	60 (4272)	67.9% (19/28)	4000 (30006000)	53% (8/15)	100% (28/28)	100 (82117)	10 (811)	28 (2630)		49.5 (3662)	50 (3855)	Pneumonia (21/50) Non-pulmonary sepsis (8/50) Aspiration pneumonia (3/50) Other shock (4/50) Drug-induced ARDS (3/50) Others (11/50)
RV INJURY ()	51 (3765)	50.0% (9/18)	4000 (20006000)	45% (16/35)	100% (18/18)	122 (86150)	8 (810)	24 (2028)		63 (5566)	46 (3562)	
*Legras/2015*												
RV INJURY (+)	56 (15)			50.0% (18/36) *	100% (36/36) *	112 (91=154)	10 (814) **			CI: 2.9 (2.63.4) ** (L/min/m^2^)	46 (17)	Pneumonia (62%) No other etiologies were reported
RV INJURY ()				47.7% (62/130) *	100% (130/130) *	114 (72145)	11 (812) **			CI: 3.2 (2.64.0) ** (L/min/m^2^)		
*Lazzeri/2016*												
RV INJURY (+)	57 (14)	70.6% (12/17)		64.7% (11/17)	100% (17/17)						44 (19)	H1N1 influenza (23/74) H3N2 (2/74) Viral pneumonia (8/74) Bacterial pneumonia (43/74) ***
RV INJURY ()	50 (15)	73.7% (42/57)		45.6% (26/57)	100% (57/57)							
*Mekontso Dessap /2016*												
RV INJURY (+)	57 (16)	63.4% (104/164)		68.,9% (113/164)	100% (164/164)	106 (40)	8 (4)	26 (5)	28 (11)		50 (19)	Pneumonia (93/164) Aspiration pneumonitis (16/164) Non-pulmonary sepsis (34/164) Others (17/164)
RV INJURY ()	58 (17)	68.5% (403/588)		66.2% (389/588)	100% (588/588)	118 (42)	8 (4)	24 (4)	32 (12)		54 (21)	Pneumonia (200/588) Aspiration pneumonitis (75/588) Non-pulmonary sepsis (228/588) Others (73/588)
*See/2017*												
RV INJURY (+)	65 (13)	65.2% (43/66)		33.3% (22/66)	89.4% (59/66)	169 (63)	7 (3)	21 (5)	34 (18)	LVEF<40% (10/66)	APACHE II: 27 (9)	Pneumonia (57/66) Non-pulmonary sepsis (9/66)
RV INJURY ()	62 (15)	61.3% (103/168)		32.7% (55/168)	88.1% (148/168)	172 (69)	6 (3)	21 (2)	29 (12)	LVEF<40% (28/168)	APACHE II: 27 (8)	Pneumonia (151/168) Non-pulmonary sepsis (17/168)
*Bonizzoli/2018*												
RV INJURY (+)	58	53.3% (16/30)			100% (3/3)	107	12.7			54.9%	43.2	Viral pneumonia (7/30) Bacterial pneumonia (23/30)
RV INJURY ()					100% (25/25)							
*Zeiton/2018*												
RV INJURY (+)	39 (14)	30.0% (3/10)		100% (10/10)	100% (10/10)	77 (13)	14 (1)	40 (7)				Pneumonia (9/10) Other causes of ARDS were unclear
RV INJURY ()	49 (15)	48.6% (17/35)		74.3% (26/35)	100% (35/35)	151 (61)	11 (2)	32 (10)				Pneumonia (17/35) Other causes of ARDS were unclear

RV injury includes RV dysfunction, RV dysfunction with hemodynamic compromise, RV failure, or acute cor-pulmonale in the studies

*The numbers were described for patients with only ACP or without ACP and PFO (Patients with only PFO or with PFO and ACP were excluded)

**The value was reported as median with interquartile range

***The summation of each number did not fit the reported total number

The standard deviation could not be calculated

The numbers and percentages are per total population included in the study but not all patients were evaluated for RVD/ACP. Therefore, the total numbers were different from actually analyzed number of patients

### Outcomes

**Fig. 2:**
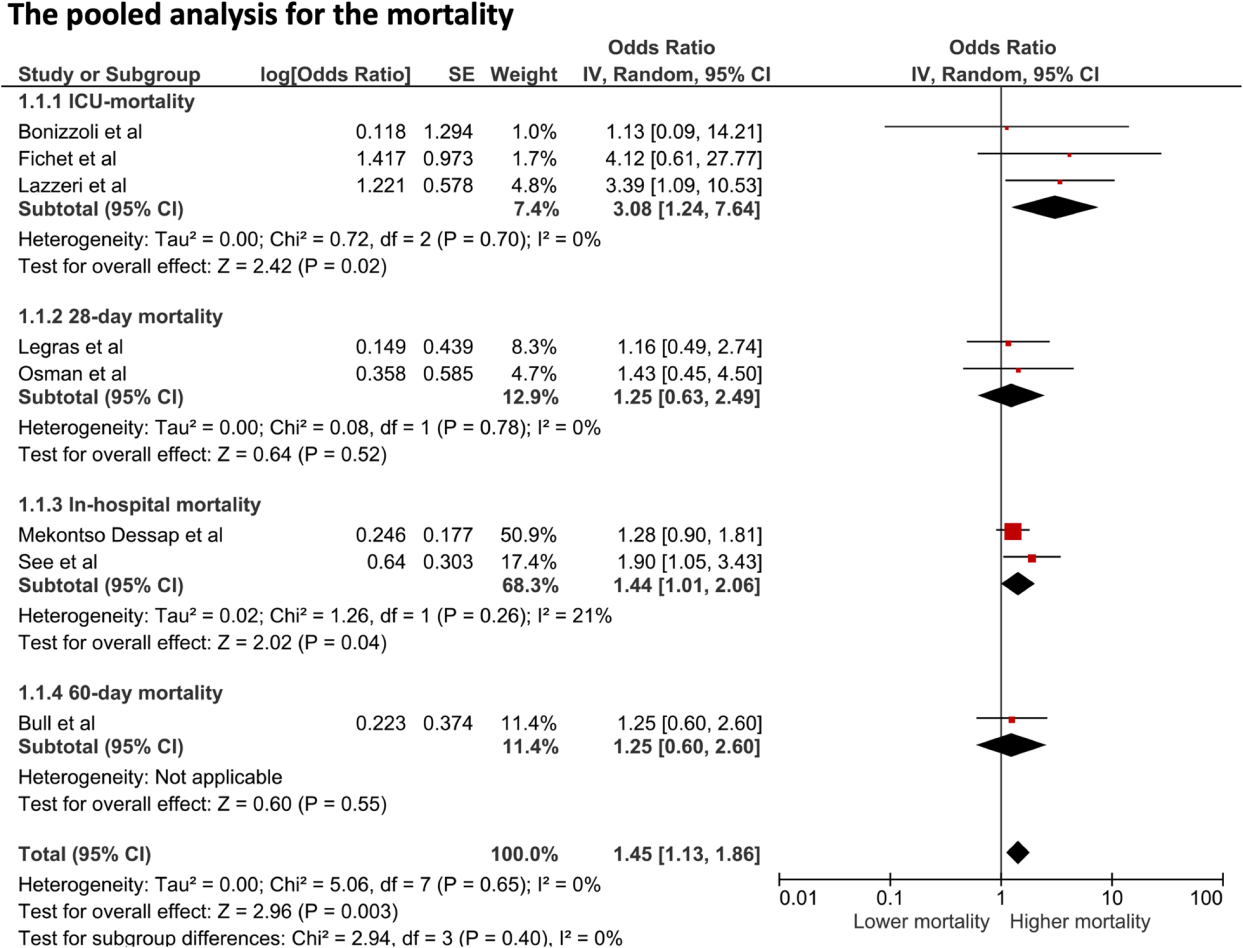
Forest plot of patients with right ventricular injury versus those without: the pooled odds ratios of ICU-mortality, 28-day mortality, In-hospital mortality, 60-day mortality, and overall mortality

RV injury that included RV dysfunction, RV dysfunction with hemodynamic compromise, RV failure, or acute cor-pulmonale was present in 21.0% (391/1,861) of the cohort. In the pooled meta-analysis of 9 studies, the presence of RV injury in patients with ARDS was associated with a significantly higher overall mortality (OR 1.45, 95% CI 1.131.86, *p*-value=0.003, *I*^2^=0%), as shown in Fig.2. In subgroup analysis investigating short-term and long-term mortalities, the presence of RV injury in patients with ARDS was associated with significantly higher short-term mortality (OR 1.48, 95% CI 1.141.93, *p*-value=0.003, *I*^2^=0%), while the association was not significant in long-term mortality (OR 1.24, 95% CI 0.662.33, *p*-value=0.003, *I*^2^=0%), as shown in Additional file4.

In the pooled analysis of 3 studies that investigated adjusted odds ratio of mortality, the presence of RV injury was associated with significantly higher mortality (OR 1.95, 95% CI 1.302.93, *p*-value=0.001, *I*^2^=0%), as shown in Additional file 4. Although Lazzeri et al. reported OR for ICU-mortality using a stepwise regression analysis adjusting for tricuspid annular plane systolic excursion (TAPSE)<16mm, we did not include this study in the pooled analysis of studies investigated adjusted OR because this was not a multivariate analysis adjusting for risk factors of ICU-mortality.

We detected no evidence of publication bias when we assessed the funnel plots visually, as shown in Additional file2. We also statistically assessed publication bias using Eggers regression test and found no publication bias (*p*-value=0.080).

## Discussion

In this systematic review and meta-analysis, that included 1,861 patients with ARDS, RV injury was present in 21.0% (391 patients) of the cohort. The presence of RV injury in ARDS was associated with a significantly higher risk of overall and short-term mortality. This result was consistent with previously reported prevalence of acute cor pulmonale in patients with ARDS [29]. Our study highlights the importance of assessment of RV in patients with ARDS and suggests that the prevention and therapeutic intervention for RV injury could be the target to improve the outcome of patients with ARDS.

This systematic review also highlights that RV injury in literature was evaluated by different modalities and a multitude of definitions which might account for the wide range (9.5% to 89.5%) of reported prevalence of RV injury in ARDS. The ideal modality for the recognition of RV injury in critically ill patients remains inconclusive [30]. Although the PAC was used to assess RV injury in earlier studies, the use of PAC has been recently declining since it was reported to be associated with increased adverse events without improving mortality [5]. TTE has been widely used in the intensive care unit to assess RV injury. However, the complex anatomy of RV and the challenges of adequate image-acquirement in patients with ARDS are major limitations of TTE. Hence, TEE may be preferred to TTE to assess RV injury [31]. However, in most ICUs expertise and access to TEE remains limited, constraining the widespread applicability of TEE as a modality of choice. In addition, as shown in our systematic review, various parameters used to define RV injury adds to inconsistency in our understanding of RV injury in ARDS. This variability arises from the lack of a standardized definition of RV injury in critically ill patients, supporting the acute need for validated criteria for RV injury in ARDS with various modalities to better understand the prevalence and impact of RV injury in patients with ARDS.

In our study, we demonstrated that RV injury in ARDS was associated with increased short-term and overall mortalities. Initial studies [16, 20, 21, 23, 27] were not conclusive in assessing the impact of RV injury in ARDS owing to their limited sample size and heterogeneity of the study population.

The determinants of higher mortality with RV injury in patients with ARDS remain poorly understood. Studies have identified driving pressure18 cmH_2_O, PaCO248 cm_2_O, and *P*/*F* ratio<150mmHg as independent factors associated with the development of RV injury [21]. In some, the compromised right ventricle enters a vicious cycle of hemodynamic compromise from cor-pulmonale, deteriorating organ perfusion and failure culminating into death. The concern for higher mortality with RV injury in ARDS has steered experts from the Lung protective to the RV protective approach in ARDS management. The management entails reducing lung stress by limiting plateau pressure<27cm H_2_O [32] and driving pressure at<18cm H_2_O [21]. In a study investigating RV injury before the widespread use of lung protective strategy, the reported prevalence of RV injury was significantly higher [33]. In this meta-analysis with included studies being conducted after prevalent use of lung-protective ventilation strategy, which is also RV-protective, the prevalence of RV injury was found to be 21%. This reiterates lung protective ventilation remains the cornerstone of RV protective strategy. Higher PEEP or permissive hypercapnia, which are routinely used in ARDS management, may need to be modified in patients at high-risk of RV injury. Prone position ventilation, an intervention with a mortality benefit in ARDS has also been shown to relieve RV enlargement and septal dyskinesia by reducing PVR [34, 35]. The use of pulmonary vasodilators or inotropic agents may also have a role in reducing PVR in RV injury [36]. Veno-venous extracorporeal membranous oxygenation or extracorporeal carbon dioxide removal has been shown to unload the RV in patients with ARDS and RV injury [37]. In addition, extracorporeal management facilitates limiting injurious ventilator settings and correcting hypercapnia, factors know to worsen RV injury. It still remains unclear if the integration of these interventions in a systematic fashion translates to improved clinical outcomes. A randomized controlled trial with well-defined criteria for the early diagnosis of RV injury is warranted to evaluate the effectiveness of the RV-protective strategy.

There are several limitations to this study. First, the sample sizes of the included studies were relatively small. However, the results of all included studies were quite consistent and the generalizability of this studies finding appears to be robust. Second, the definition and modality used to define RV injury were not consistent and this might have affected the result of each study. In addition to inconsistent criteria, the limited information of loading conditions including PEEP, plateau pressure, and fluid balance made it challenging to assess RV function accurately. Future studies evaluating RV injury in critically ill patients need to use validated criteria developed in concordance with existing American Society of Echocardiography guidelines to ensure consistent reporting of prevalence and outcomes of RV injury in this population. As of now, the Preferred Reporting Items for Critical care Echocardiography Studies (PRICES) project endorsed by the European Society of Intensive Care Medicine has been published [38, 39]. In this recommendation, RV fraction area change, RV S tissue doppler imaging, TAPSE, RVEDA, RVED diameter, RVEDA/LVEDA, tricuspid regurgitation peak velocity, and/or pulmonary artery pressures are considered to be essential items to report RV function. Of these, further standardization of assessment of RV function is warranted. Third, only two studies investigated long-term mortality and the association between the presence of RV injury and long-term mortality was not significant [23, 27]. In addition to a small number of included patients, this might be also because long-term mortality in patients with ARDS mainly depends on non-modifiable factors such as age or comorbidities while short-term outcome has improved with the development of therapeutic interventions [40].

## Conclusion

In this systematic review and meta-analysis including 1,861 patients with ARDS, the presence of RV injury was significantly associated with increased overall and short-term mortality. This result implicates the importance of right ventricle assessments in patients with ARDS.

## Supplementary Information

**Additional file 1**.The search strategy.

**Additional file2**. Funnel plot analysis of publication bias of Short-term mortality.

**Additional file3**.Inclusion and exclusion criteria of each study.

**Additional file 4**. Pooled analysis of adjusted odds ratio for mortality, short term mortality and long term mortality in ARDS with and without RV dysfunction.

## Data Availability

All data associated with this manuscript are included in the main text and supplementary materials.

## Abbreviations

RV: Right ventricle/right ventricular
RV: Right ventricle/right ventricular
ARDS: Acute respiratory distress syndrome
ICU: Intensive care unit
TTE: Transthoracic echocardiography
TEE: Transesophageal echocardiography
PRISMA: Preferred reporting items for systematic reviews and meta-analyses
OR: Odds ratio
CIs: Confidence intervals
PEEP: Positive end-expiratory pressure
RVEDA: Right ventricular end-diastolic area
LVEDA: Left ventricular end-diastolic area
TAPSE: Tricuspid annular plane systolic excursion
St: Peak systolic velocity at the tricuspid valve
ACP: Acute cor pulmonale
VV-ECMO: Veno-venous extracorporeal membrane oxygenation
MPAP: Mean pulmonary artery pressure
RCT: Randomized controlled trial
PAC: Pulmonary artery catheter
MPAP: Mean pulmonary artery occlusion pressure
CVP: Central venous pressure
PAOP: Pulmonary artery occlusion pressure
SVI: Stroke volume index

## References

[1] Zhang Z, Spieth PM, Chiumello D, Goyal H, Torres A, Laffey JG, Hong Y (2019). Declining mortality in patients with acute respiratory distress syndrome: an analysis of the acute respiratory distress syndrome network trials. Crit Care Med.

[2] Zochios V, Parhar K, Tunnicliffe W, Roscoe A, Gao F (2017). The right ventricle in ARDS. Chest.

[3] Price LC, McAuley DF, Marino PS, Finney SJ, Griffiths MJ, Wort SJ (2012). Pathophysiology of pulmonary hypertension in acute lung injury. Am J Physiol Lung Cell Mol Physiol.

[4] Mahmood SS, Pinsky MR (2018). Heart-lung interactions during mechanical ventilation: the basics. Ann Transl Med.

[5] Investigators TE, Coordinators ES (2005). Evaluation study of congestive heart failure and pulmonary artery catheterization effectiveness. The ESCAPE trial. JAMA.

[6] Liberati A, Altman DG, Tetzlaff J, Mulrow C, Gotzsche PC, Ioannidis JP, Clarke M, Devereaux PJ, Kleijnen J, Moher D (2009). The PRISMA statement for reporting systematic reviews and meta-analyses of studies that evaluate health care interventions: explanation and elaboration. J Clin Epidemiol.

[7] Moher D, Shamseer L, Clarke M, Ghersi D, Liberati A, Petticrew M, Shekelle P, Stewart LA, Group P-P (2015). Preferred reporting items for systematic review and meta-analysis protocols (PRISMA-P) 2015 statement. Syst Rev.

[8] Stroup DF, Berlin JA, Morton SC, Olkin I, Williamson GD, Rennie D, Moher D, Becker BJ, Sipe TA, Thacker SB (2000). Meta-analysis of observational studies in epidemiology: a proposal for reporting. Meta-analysis of observational studies in epidemiology (MOOSE) group. JAMA.

[9] Bernard GR, Artigas A, Brigham KL, Carlet J, Falke K, Hudson L, Lamy M, Legall JR, Morris A, Spragg R (1994). The American-European consensus conference on ARDS. Definitions, mechanisms, relevant outcomes, and clinical trial coordination. Am J Respir Crit Care Med.

[10] Force TADT (2012). Acute respiratory distress syndrome: the Berlin definition. JAMA.

[11] DerSimonian R, Laird N (1986). Meta-analysis in clinical trials. Control Clin Trials.

[12] Higgins JP, Thompson SG, Deeks JJ, Altman DG (2003). Measuring inconsistency in meta-analyses. BMJ.

[13] Easterbrook PJ, Berlin JA, Gopalan R, Matthews DR (1991). Publication bias in clinical research. Lancet.

[14] Stang A (2010). Critical evaluation of the Newcastle-Ottawa scale for the assessment of the quality of nonrandomized studies in meta-analyses. Eur J Epidemiol.

[15] Boissier F, Katsahian S, Razazi K, Thille AW, Roche-Campo F, Leon R, Vivier E, Brochard L, Vieillard-Baron A, Brun-Buisson C (2013). Prevalence and prognosis of cor pulmonale during protective ventilation for acute respiratory distress syndrome. Intensive Care Med.

[16] Bonizzoli M, Cipani S, Lazzeri C, Chiostri M, Ballo P, Sarti A, Peris A (2018). Speckle tracking echocardiography and right ventricle dysfunction in acute respiratory distress syndrome a pilot study. Echocardiography (Mount Kisco, NY).

[17] Lazzeri C, Bonizzoli M, Cianchi G, Batacchi S, Chiostri M, Peris A (2020). Severity of acute respiratory distress syndrome and echocardiographic findings in clinical practice-an echocardiographic pilot study. Heart Lung.

[18] Lazzeri C, Bonizzoli M, Cianchi G, Batacchi S, Chiostri M, Fulceri G, Peris A (2020). Right ventricular hypertrophy in refractory acute respiratory distress syndrome treated with venovenous extracorporeal membrane oxygenation support. J Cardiothorac Vasc Anesth.

[19] Lazzeri C, Cianchi G, Bonizzoli M, Batacchi S, Terenzi P, Bernardo P, Valente S, Gensini GF, Peris A (2016). Right ventricle dilation as a prognostic factor in refractory acute respiratory distress syndrome requiring veno-venous extracorporeal membrane oxygenation. Minerva Anestesiol.

[20] Legras A, Caille A, Begot E, Lhritier G, Lherm T, Mathonnet A, Frat JP, Courte A, Martin-Lefvre L, Goullo JP (2015). Acute respiratory distress syndrome (ARDS)-associated acute cor pulmonale and patent foramen ovale: a multicenter noninvasive hemodynamic study. Crit Care (London, England).

[21] Mekontso Dessap A, Boissier F, Charron C, Bgot E, Repess X, Legras A, Brun-Buisson C, Vignon P, Vieillard-Baron A (2016). Acute cor pulmonale during protective ventilation for acute respiratory distress syndrome: prevalence, predictors, and clinical impact. Intensive Care Med.

[22] amendys-Silva SA, Santos-Martnez LE, Pulido T, Rivero-Sigarroa E, Baltazar-Torres JA, Domnguez-Cherit G, Sandoval J (2014). Pulmonary hypertension due to acute respiratory distress syndrome. Braz J Med Biol Res.

[23] Osman D, Monnet X, Castelain V, Anguel N, Warszawski J, Teboul JL, Richard C (2009). Incidence and prognostic value of right ventricular failure in acute respiratory distress syndrome. Intensive Care Med.

[24] See KC, Ng J, Siow WT, Ong V, Phua J (2017). Frequency and prognostic impact of basic critical care echocardiography abnormalities in patients with acute respiratory distress syndrome. Ann Intensive Care.

[25] Vieillard-Baron A, Schmitt JM, Augarde R, Fellahi JL, Prin S, Page B, Beauchet A, Jardin F (2001). Acute cor pulmonale in acute respiratory distress syndrome submitted to protective ventilation: incidence, clinical implications, and prognosis. Crit Care Med.

[26] Zeiton TM, Elsayed HEM, Hassan OS, Sarhan AM (2018). Clinical risk score for the diagnosis of acute cor pulmonale in acute respiratory distress syndrome. Egypt J Chest Dis Tuberculosis.

[27] Bull TM, Clark B, McFann K, Moss M (2010). Pulmonary vascular dysfunction is associated with poor outcomes in patients with acute lung injury. Am J Respir Crit Care Med.

[28] Fichet J, Moreau L, Gene O, Legras A, Mercier E, Garot D, Dequin PF, Perrotin D (2012). Feasibility of right ventricular longitudinal systolic function evaluation with transthoracic echocardiographic indices derived from tricuspid annular motion: a preliminary study in acute respiratory distress syndrome. Echocardiography (Mount Kisco, NY).

[29] Das SK, Choupoo NS, Saikia P, Lahkar A (2017). Incidence proportion of acute cor pulmonale in patients with acute respiratory distress syndrome subjected to lung protective ventilation: a systematic review and meta-analysis. Indian J Crit Care Med.

[30] Dugar SP, Vallabhajosyula S (2020). Right ventricle in sepsis: clinical and research priority. Heart.

[31] Lhritier G, Legras A, Caille A, Lherm T, Mathonnet A, Frat JP, Courte A, Martin-Lefvre L, Goullo JP, Amiel JB (2013). Prevalence and prognostic value of acute cor pulmonale and patent foramen ovale in ventilated patients with early acute respiratory distress syndrome: a multicenter study. Intensive Care Med.

[32] Jardin F, Vieillard-Baron A (2007). Is there a safe plateau pressure in ARDS? The right heart only knows. Intensive Care Med.

[33] Jardin F, Gueret P, Dubourg O, Farcot JC, Margairaz A, Bourdarias JP (1985). Two-dimensional echocardiographic evaluation of right ventricular size and contractility in acute respiratory failure. Crit Care Med.

[34] Jozwiak M, Teboul JL, Anguel N, Persichini R, Silva S, Chemla D, Richard C, Monnet X (2013). Beneficial hemodynamic effects of prone positioning in patients with acute respiratory distress syndrome. Am J Respir Crit Care Med.

[35] Gurin C, Reignier J, Richard J-C, Beuret P, Gacouin A, Boulain T, Mercier E, Badet M, Mercat A, Baudin O (2013). Prone positioning in severe acute respiratory distress syndrome. N Engl J Med.

[36] Ventetuolo CE, Klinger JR (2014). Management of acute right ventricular failure in the intensive care unit. Ann Am Thorac Soc.

[37] Reis Miranda D, van Thiel R, Brodie D, Bakker J (2015). Right ventricular unloading after initiation of venovenous extracorporeal membrane oxygenation. Am J Respir Crit Care Med.

[38] Huang S, Sanfilippo F, Herpain A, Balik M, Chew M, Clau-Terr F, Corredor C, De Backer D, Fletcher N, Geri G (2020). Systematic review and literature appraisal on methodology of conducting and reporting critical-care echocardiography studies: a report from the European Society of Intensive Care Medicine PRICES expert panel. Ann Intensive Care.

[39] Sanfilippo F, Huang S, Herpain A, Balik M, Chew MS, Clau-Terr F, Corredor C, De Backer D, Fletcher N, Geri G (2021). The PRICES statement: an ESICM expert consensus on methodology for conducting and reporting critical care echocardiography research studies. Intensive Care Med.

[40] Chiumello D, Coppola S, Froio S, Gotti M (2016). What's next after ARDS: long-term outcomes. Respir Care.

