# Supplementary material for: The impact of right ventricular injury on the mortality in patients with acute respiratory distress syndrome: a systematic review and meta-analysis
Source: Crit Care. 2021 May 21;25:172. doi: 10.1186/s13054-021-03591-9 (PMC8138512; doi:10.1186/s13054-021-03591-9)
Supplement: Supplementary file 1 — Additional file 1.The search strategy. [file 13054_2021_3591_MOESM1_ESM.docx]

**Supplementary Table 1: The search strategy.**

**Ovid MEDLINE® ALL 1946 to August 28, 2020**

1 Respiratory Distress Syndrome, Adult/ or (adult respiratory distress syndrome or acute respiratory distress syndrome or ARDS).mp.

2 exp Respiratory Insufficiency/ or (acute respiratory failure or respiratory insufficiency).mp.

3 exp Acute Lung Injury/ or acute lung injury.mp.

4 or/1-3

5 Ventricular Function, Right/

6 Ventricular Dysfunction, Right/

7 (right ventric* adj5 (failure or function or dysfunction)).mp.

8 (right heart adj5 (failure or function or dysfunction)).mp.

9 Pulmonary Heart Disease/ or pulmonary heart disease.mp.

10 cor pulmonale.mp.

11 or/5-10

12 4 and 11

13 limit 12 to yr="1990 -Current"

**Embase 1974 to 2020 August 28**

1 adult respiratory distress syndrome/ or (adult respiratory distress syndrome or acute respiratory distress syndrome or ARDS).mp.

2 exp respiratory failure/ or (acute respiratory failure or respiratory insufficiency).mp.

3 exp acute lung injury/ or acute lung injury.mp.

4 or/1-3

5 heart right ventricle function/

6 heart right ventricle failure/

7 (right ventric* adj5 (failure or function or dysfunction)).mp.

8 (right heart adj5 (failure or function or dysfunction)).mp.

9 cor pulmonale/ or cor pulmonale.mp.

10 pulmonary heart disease.mp.

11 or/5-10

12 4 and 11

13 conference abstract.pt.

14 12 not 13

15 limit 14 to yr="1990 -Current"

**Cochrane Central Register of Controlled Trials**

**Issue 8 of 12, August 2020**

#1 MeSH descriptor: [Respiratory Distress Syndrome, Adult] this term only

#2 ("adult respiratory distress syndrome" OR "acute respiratory distress syndrome" OR ARDS):ti,ab,kw

#3 MeSH descriptor: [Respiratory Insufficiency] explode all trees

#4 ("acute respiratory failure" OR "respiratory insufficiency"):ti,ab,kw

#5 MeSH descriptor: [Acute Lung Injury] explode all trees

#6 ("acute lung injury"):ti,ab,kw

#7 {OR #1-#6}

#8 MeSH descriptor: [Ventricular Function, Right] this term only

#9 MeSH descriptor: [Ventricular Dysfunction, Right] this term only

#10 (right ventric* NEAR/5 (failure or function or dysfunction)):ti,ab,kw

#11 (right heart NEAR/5 (failure or function or dysfunction)):ti,ab,kw

#12 MeSH descriptor: [Pulmonary Heart Disease] this term only

#13 ("pulmonary heart disease"):ti,ab,kw

#14 ("cor pulmonale"):ti,ab,kw

#15 {OR #8-#14}

#16 #7 AND #15 with Publication Year from 1990 to 2020, in Trials
