# Supplementary figures and images for: The impact of right ventricular injury on the mortality in patients with acute respiratory distress syndrome: a systematic review and meta-analysis
Source: Crit Care. 2021 May 21;25:172. doi: 10.1186/s13054-021-03591-9 (PMC8138512; doi:10.1186/s13054-021-03591-9)

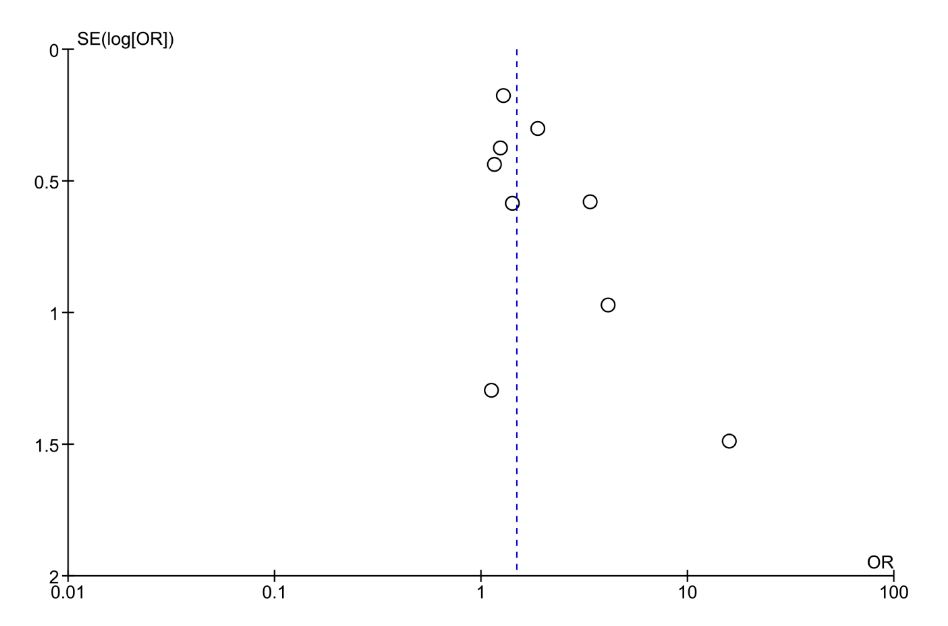

Supplement: Supplementary file 2 — Additional file2. Funnel plot analysis of publication bias of Short-term mortality. [file 13054_2021_3591_MOESM2_ESM.jpg]

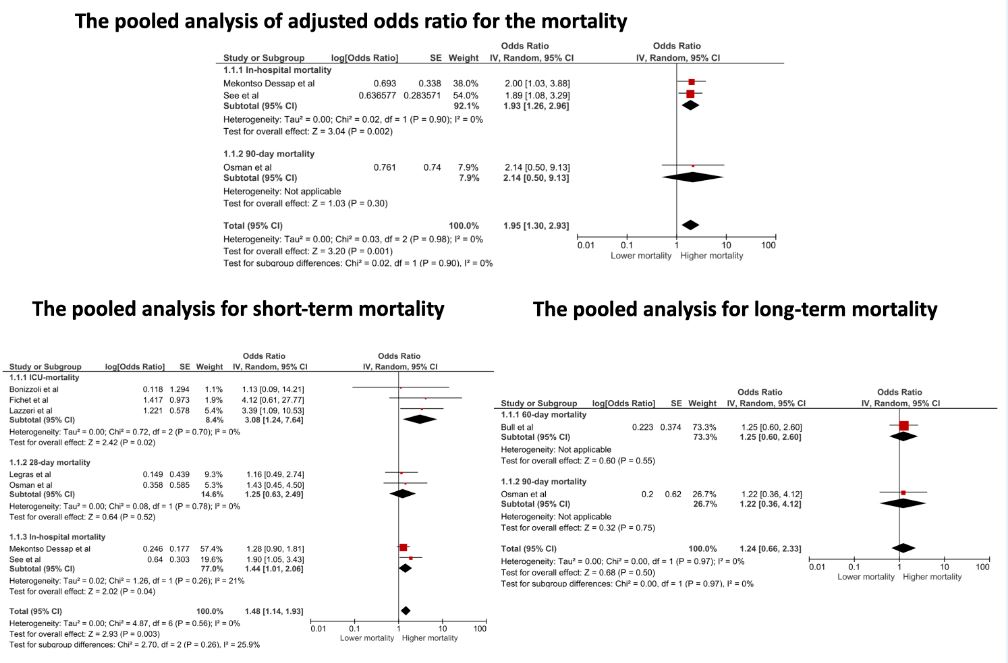

Supplement: Supplementary file 4 — Additional file 4. Pooled analysis of adjusted odds ratio for mortality, short term mortality and long term mortality in ARDS with and without RV dysfunction. [file 13054_2021_3591_MOESM4_ESM.jpg]
