# Supplementary material for: The impact of right ventricular injury on the mortality in patients with acute respiratory distress syndrome: a systematic review and meta-analysis
Source: Crit Care. 2021 May 21;25:172. doi: 10.1186/s13054-021-03591-9 (PMC8138512; doi:10.1186/s13054-021-03591-9)
Supplement: Supplementary file 3 — Additional file3.Inclusion and exclusion criteria of each study. [file 13054_2021_3591_MOESM3_ESM.docx]

**Supplementary Table 2: Inclusion and exclusion criteria of each study.**

| **Authors** | **Inclusion criteria** | **Exclusion criteria** |
| --- | --- | --- |
| **Bonizzoli / 2018** | - Moderate-severe ARDS based on Berlin definition | - Not reported |
| **Bull / 2010** | - ARDS based on American European Consensus Conference diagnostic criteria. - Patients who were intubated and received positive pressure ventilation. | - The presence of pulmonary artery catheter after the onset of acute lung injury. - The presence of acute lung injury for more than 48 hours. - Inability to obtain consent. - The presence of chronic conditions that could independently influence survival, impair weaning, or compromise compliance with the protocol. - Irreversible conditions for which the estimated six-month mortality rate exceeded 50 percent. |
| **Fichet / 2012** | - Mechanical ventilation initiated in the last 48 hours, in controlled mode, without spontaneous breathing. - P/F ratio < 200 for more than 12 hours with recent pulmonary infiltrates and no suspicion of cardiogenic edema. | - Chronic respiratory failure. - Previous history of right ventricular failure. - Left ventricular ejection fraction ≤ 35% or significant valvular disease. |
| **Lazzeri / 2016** | - Refractory ARDS requiring VV-ECMO. | - Not reported. |
| **Legras / 2015** | - ARDS based on American European Consensus Conference diagnostic criteria modified for one criterion (P/F ratio ≤ 200 with a FiO_2_ of 1 and PEEP ≥ 5cmH_2_O). | - Not reported. |
| **Mekonstso Dessap / 2016** | - Moderate-severe ARDS based on Berlin definition. - Patients underwent transesophageal echocardiography (TEE) within 3 days following the diagnosis of ARDS. | - Contraindications for TEE (esophageal diseases or major uncontrolled bleeding). - The presence of a chronic pulmonary disease requiring long-term oxygen therapy or home mechanical ventilation. |
| **Osman / 2009** | - ARDS based on requiring mechanical ventilation for more than 24 hours. - Patients who underwent pulmonary artery catheter placement within 2 hours from randomization. | - Patients younger than 18 years. - Patients with hemorrhagic shock. - Patients with myocardial infarction complicated by cardiogenic shock requiring revascularization. - Thrombocytopenia (≤ 10.0 x 10^9^/L). - Participation in other trials within the last 30 days. - Patients who were moribund. - If patients’ physician declined the use of full life support. |
| **See / 2017** | - Patients with ARDS who underwent basic critical care echocardiography within 48 hours of admission. | - Patients who had surgical dressing over the chest/abdomen precluding satisfactory transthoracic echocardiographic windows. |
| **Zeiton / 2018** | - Older than 18 years old - ARDS based on Berlin definition who are mechanically ventilated/ - Transthoracic echocardiography was performed within 72 hours of ARDS diagnosis. | - Patients who refused to be involved. - Pregnancy. - Previous history of pulmonary hypertension. - Previous history of pulmonary embolism. - Previous history of deep venous thrombosis. - Previous history of chronic pulmonary disease requiring long-term oxygen therapy or home mechanical ventilation. - Patients in prone positioning. |
